# Chlorate of Potash in Ulcerative Stomatitis, Etc.

**Published:** 1853-08

**Authors:** 


From the American Jour. of Med. Science.

*Chlorate of Potash in Ulcerative Stomatitis and Cancrum Oris.*

Dr. J. H. Babington states (*Dublin Quarterly Journal*, Feb. 1853,) that, in an epidemic of ulcerative stomatitis which occurred in the Coleraine Union Workhouse, in 1848, he used the chlorate of potash with great success. The treatment adopted was a mild aperient of rhubarb and magnesia, and the administration of chlorate of potash, dissolved in water, sweetened with syrup, in doses of four grains, every fourth hour; the mouth was also washed with a weak lotion of solution of chloride of soda. They all recovered in about six days. Dr. B. treated one case with alteratives and tonics, and it was three weeks before it got quite well; thereby proving the efficacy of the chlorate of potash.

